# Life-Threatening Obstructive Sleep Apnea Caused by Adenoid Hypertrophy in an Infant with Noonan Syndrome

**DOI:** 10.1155/2012/514514

**Published:** 2012-11-05

**Authors:** Sonia Khirani, Nicolas Leboulanger, Adriana Ramirez, Brigitte Fauroux

**Affiliations:** ^1^IP Santé, 94200 Ivry sur Seine, France; ^2^Pediatric Pulmonary Department, AP-HP, Hôpital Armand Trousseau, 75012 Paris, France; ^3^Pediatric Head and Neck Surgery Department, AP-HP, Hôpital Armand Trousseau, 75012 Paris, France; ^4^Université Pierre et Marie Curie-Paris 6, 75012 Paris, France; ^5^ADEP Assistance, 92150 Suresnes, France; ^6^Université Pierre et Marie Curie-Paris 6 and INSERM U 955, 94000 Créteil, France

## Abstract

Adenoidectomy is a commonly performed surgery in children, even though its effectiveness is still under investigation. However, in children with risk factors such as age under 3 years old, associated comorbidities, or severe obstructive sleep apneas, a high postoperative respiratory morbidity is possible. We report the case of a 15-month-old boy with Noonan syndrome and a complex clinical history, who presented with a life-threatening obstructive sleep apnea due to hypertrophy of the adenoids which resolved completely after adenoidectomy.

## 1. Introduction

Adenoidectomy is a common procedure in children with obstructive sleep apnea (OSA). However, in children with risk factors such as age under 3 years old, associated comorbidities, or severe OSA, a high postoperative respiratory morbidity is possible.

Indications for adenoidectomy in children include recurrent or chronic nasal discharge, recurrent episodes of acute otitis media, persistent otitis media with effusion, and symptoms of upper airway obstruction [1]. Adenoidectomy is one of the most frequently surgical procedures performed in western countries children [1] and is considered as an uncomplicated surgery at low risk of respiratory morbidity [2]. However, its effectiveness is still under investigation [1, 3]. 

Postoperative respiratory complications may occur more frequently if specific risk factors are present such as age lower than 3 years old and comorbidities such as airway anomalies, heart diseases, significant craniofacial anomalies, hypotonia, and severe OSA as indicated by repetitive decreases in oxyhemoglobin saturation (SpO_2_) <80% [4, 5]. Moreover, a high apnea-hypopnea index and repetitive episodes of profound hypoxemia during sleep increase the risk for postadenotonsillectomy respiratory morbidity at least 20-fold [2]. 

Noonan syndrome is a relatively common congenital genetic disorder with an estimated prevalence of 1 in 1000 to 2500 live births [6], characterized by distinctive facial deformities, short stature, chest deformity, congenital heart disease, and other associated conditions. The main facial features of Noonan syndrome are hypertelorism with downslanting palpebral fissures, ptosis, and low-set posteriorly rotated ears with a thickened helix [7, 8]. The cardiovascular defects usually associated with this condition are pulmonary stenosis and hypertrophic cardiomyopathy. Additional associated features are possible such as a webbed neck, chest deformity, mild intellectual deficit, cryptorchidism, poor feeding in infancy, bleeding tendency, and lymphatic dysplasias. 

We report here the case of an infant with Noonan syndrome who presented life-threatening OSA caused by hypertrophy of the adenoids, which resolved completely after adenoidectomy.

## 2. Case Report

A 15-month-old boy with Noonan syndrome was referred to our pediatric pulmonary department to assess the need for noninvasive positive pressure ventilation (NPPV). The patient was born with a body weight of 3675 g despite a 36 gestational age. He required hospital care during the first 3 weeks of life because of transient upper airway obstruction requiring nasal continuous positive airway pressure (CPAP). He was discharged home with nasal oxygen therapy for a few weeks. He also presented a moderated hypertrophic cardiomyopathy treated with beta-blocker therapy that was stopped after 6 months after improvement on ultrasonographic examination. 

Clinically, the patient presented with facial anomalies including short neck, micrognathia, macroglossia with glossoptosis, narrowed choanes, low-set ears, and a thoracic deformity (“cask” thorax). He needed nutritional support by nocturnal nasogastric tube because of swallowing and feeding difficulties. Axial and peripheral hypotonia was present during the first month of life but improved with age. 

At the age of 6 months, he was hospitalized during several weeks for an important persistent dyspnea due to rhinitis and major nasal obstruction and at the age of 12 months for a pneumopathy that rapidly responded to oral antibiotics. He was hospitalized again at the age of 15 months because of important desaturations during his daytime nap. Clinical examination was normal during wakefulness, while numerous obstructive apneas, with repeated oxyhemoglobin desaturation (with SpO_2_ <75%) followed by frequent awakenings and sweating, were observed during daytime naps. Nocturnal sleep study was therefore undergone and confirmed the previous findings. Morning venous blood gases were measured and showed a pH value of 7.34, a partial venous carbon dioxide tension (PvCO_2_) of 60 mmHg and bicarbonates at 31.5 mmol/L. The patient was discharged home as he was clinically stable. It was noticed that obstructive symptoms improved when the patient slept on supine position.

On the consecutive respiratory examination in our department, rhinitis was diagnosed with the presence of slight fever and major mucous secretions. Diurnal blood gas measurements, as well as nocturnal blood gases assessment (with oxygen therapy at 1 L/min), were performed (SenTec AG, Therwil, Switzerland). We found altered daytime blood gases and nocturnal hypercapnia (Table 1). The movement and fragmentation index (MFI) and sleep efficiency were measured by overnight actigraphy (Actiwatch, Cambridge Neurotechnology Ltd., Cambridge, UK). MFI was defined as the sum of the minutes of movement expressed as a percentage of the total recording time (movement index), and the number of times that sleep was terminated after 1 minute expressed as a percentage of the total estimated sleep time (fragmentation index). The sum is expressed as a percentage of the duration of the nocturnal recording and is used as an index of restlessness. Thus, the higher the index, the greater the sleep disruption. Sleep efficiency was defined as the percentage of time spent asleep whilst in bed. MFI was abnormally high with a value of 83%, and sleep efficiency was low (59%). Clinical and fiberoptic examination showed a hypertrophy of the adenoids which completely obstructed the nasopharynx and normal tonsils. Adenoidectomy was promptly performed. No complication was observed during or after the surgical procedure.

Two months after surgery, all symptoms of OSA had resolved, except for moderate sweating during the daytime nap. Diurnal blood gases and nocturnal gas exchange improved markedly (Table 1). Actigraphy showed a normal MFI of 28% and a normal sleep efficiency of 90%. Four months after surgery, the patient was eupneic and could be withdrawn from nutrition support. Daytime nap polysomnography was normal with an apnea-hypopnea index of 1 event/hour. He also considerably improved from a neurological point of view, with constant progress even though a bilateral fatigable clonus was present.

## 3. Discussion

This case report shows that adenoid hypertrophy may cause life-threatening OSA in a child with Noonan syndrome and could be cured by adenoidectomy.

As already known, OSA syndrome may negatively affect growth, neurocognitive function, and cardiovascular physiology. Many reports have suggested that children with OSA may present neurocognitive impairments, such as poor learning, behavioral problems, and attention deficit hyperactivity disorders. Finally, it is important to remind that severe untreated OSA can result in serious morbidity and even death. Gozal [9] was the first to objectively evaluate the effect of sleep-disordered breathing on intellectual function. Gozal found that a remarkably high proportion of first-grade students who had home studies suggestive of sleep-disordered breathing were performing in the lowest 10th percentile of their class academically. Moreover, children treated with tonsillectomy and adenoidectomy had a significant improvement in their grades the following year, whereas untreated children showed no change.

However, adenoids hypertrophy is alone seldom responsible for severe OSA syndrome as observed in our patient. Once the diagnosis established and because of the severity of the OSA, surgery was performed without waiting for the hypothetical results of a medical management [3]. It is also likely that the facial features of our patient increased the obstruction caused by the adenoids, worsening the OSA. Indeed, in many children with craniofacial disorders, soft tissue correction, such as adenoidectomy, does not always fully remedy the airway obstruction. Many of these children may require additional facial skeletal surgeries such as mandibular advancement, bimaxillary advancement, or even tracheotomy to bypass the airway obstruction.

The majority of children with Noonan syndrome will grow up and function normally in adulthood—they finish high school and have paying jobs—and most adults with Noonan do not require special medical care [7, 8]. So, even though guidelines recommend major precautions for adenoidectomy in patients with severe underlying conditions like our patient [1, 5, 10, 11], the risk of subsequent complications due to the presence of severe nasal obstruction and a complex clinical history in a patient with Noonan syndrome outweighs the risk of perioperative and/or postoperative complications.

In conclusion, hypertrophy of the adenoids may cause life-threatening OSA in young children with Noonan syndrome underlying the value of a systematic and regular upper airway examination and an adenoidectomy in case of OSA.

## Figures and Tables

**Table 1:** Diurnal and nocturnal gases exchanges and sleep actigraphy before and 2 months after adenoidectomy.

	Before surgery	After surgery
Diurnal gas exchange		
pH	7.39	7.40
PaO_2_ (mmHg)	57	76
PaCO_2_ (mmHg)	53	41

Nocturnal gas exchange		
Clinical condition	O_2_ 1 L·min^−1^	Ambient air
Minimal SpO_2_ (%)	79	77
Mean SpO_2_ (%)	98	97
% time spent with SpO_2_ <90% (%)	1	2
Maximal PtcCO_2_ (mmHg)	79	47
Mean PtcCO_2_ (mmHg)	59	38
% time spent with PtcCO_2_ >50 mmHg (%)	77	0

Actigraphy		
Sleep Efficiency (%)	59	90
MFI (%)	83	28

## Abbreviations

PaO_2_: Partial arterial oxygen tension
PaCO_2_: Partial arterial carbon dioxide tension
SpO_2_: Pulse oximetry
PtcCO_2_: Transcutaneous carbon dioxide tension
MFI: Movement and fragmentation index.
